# Impact of Germline *BRCA* Mutation Status on Antitumor Activity of Modified FOLFIRINOX in Patients With Advanced Pancreatic Cancer: An Exploratory Analysis

**DOI:** 10.1200/PO-25-01104

**Published:** 2026-03-05

**Authors:** Lingaku Lee, Yuichi Tachibana, Susumu Matsuo, Yusuke Niina, Takashi Fujiyama, Terumasa Hisano, Rie Sugimoto, Tetsuro Akashi, Keiichiro Ogoshi, Masayuki Furukawa

**Affiliations:** ^1^ Department of Hepato-Biliary-Pancreatology, NHO Kyushu Cancer Center, Fukuoka, Japan; ^2^ Department of Hepato-Biliary-Pancreatic Medicine, Saiseikai Fukuoka General Hospital, Fukuoka, Japan; ^3^ Department of Gastroenterology, NHO Fukuoka Higashi Medical Center, Fukuoka, Japan

## Abstract

**PURPOSE:**

Germline *BRCA* (g*BRCA*) mutations confer increased sensitivity to platinum-based chemotherapy and have been associated with improved survival across several malignancies. This study investigated the effect of g*BRCA* mutation status on the antitumor activity of modified fluorouracil, folinic acid, irinotecan, and oxaliplatin (FOLFIRINOX; mFFX) in patients with advanced pancreatic cancer (APC).

**METHODS:**

This multicenter retrospective study was performed in 178 patients with histologically confirmed APC who received mFFX, comprising 17 g*BRCA*-positive and 161 g*BRCA*-negative individuals. Efficacy outcomes, including progression-free survival (PFS), objective response rate (ORR), and normalization rate of carbohydrate antigen 19-9 (CA19-9), were compared between groups using univariate and multivariate analyses.

**RESULTS:**

Median PFS was significantly longer in g*BRCA*-positive than in g*BRCA*-negative patients (10.6 *v* 5.3 months, *P* = .005). Independent predictors of PFS included g*BRCA* mutation (hazard ratio [HR], 3.65), disease extent (HR, 2.34), and previous chemotherapy (HR, 1.66). g*BRCA-*positive patients demonstrated a significantly higher ORR (82% *v* 34%, odds ratio, 0.11, *P* < .001) and CA19-9 normalization rate (69% *v* 10%, *P* < 0.01). ORR was associated with g*BRCA* status (HR, 0.04), disease extent (HR, 0.11), and treatment history (HR, 0.27). Among 68 responders, sustained shrinkage of both primary and metastatic tumors and continued CA19-9 decline were observed during the first 6 months in g*BRCA-*positive patients, whereas responses in g*BRCA*-negative patients plateaued after 3 months. Subgroup analysis revealed that most patient subgroups demonstrated superior PFS and ORR in the g*BRCA*-positive cohort compared with those in the g*BRCA*-negative cohort.

**CONCLUSION:**

g*BRCA* mutation status was strongly associated with enhanced radiologic and biological responses to mFFX in APC. Routine screening for g*BRCA* mutations may provide essential guidance in therapeutic decision making and substantially improve clinical outcomes in this population.

## INTRODUCTION

Pancreatic cancer represents a highly lethal malignancy, characterized by both high morbidity and mortality rates.^1^ Despite recent advances in systemic and local therapies for advanced pancreatic cancer (APC), long-term survival outcomes remain poor.^2,3^ Consequently, the identification of patient subgroups that exhibit enhanced responsiveness to specific therapeutic strategies is critical for improving overall treatment efficacy and survival in this population.

**CONTEXT**

**Key Objective**
Germline *BRCA* (g*BRCA*) mutation is associated with increased platinum sensitivity in advanced pancreatic cancer (APC); however, do the kinetics of antitumor activity of modified fluorouracil, leucovorin, irinotecan, and oxaliplatin (FOLFIRINOX; mFFX) differ between g*BRCA*-positive and g*BRCA*-negative responders?
**Knowledge Generated**
Superiority of g*BRCA* mutation in patients with APC treated with mFFX was characterized by prolonged progression-free survival and pronounced radiologic and biological response. Among responders, g*BRCA*-positive patients showed sustained tumor shrinkage and carbohydrate antigen 19-9 decline during the first 6 months, whereas responses in g*BRCA*-negative patients plateaued early after 3 months.
**Relevance**
Routine testing for g*BRCA* mutation in all patients with APC is highly recommended, which can drastically change the therapeutic strategies and substantially improve therapeutic outcomes.

Loss of function in homologous recombination repair–related genes—most notably *BRCA1* and *BRCA2*—results in homologous recombination deficiency (HRD), a molecular phenotype that confers heightened sensitivity to DNA-damaging and cross-linking agents.^4,5^ Accumulating evidence demonstrates a significant survival advantage with platinum-based chemotherapy in patients with *BRCA*-associated pancreatic cancer.^6-9^ Although germline *BRCA* (g*BRCA*) mutations occur infrequently, with an estimated prevalence of only 3%-7% among patients with pancreatic cancer,^10-12^ this subset represents a biologically and clinically distinct group. Accordingly, comprehensive screening for HRD-related mutations is now strongly recommended for all patients with pancreatic cancer.^13^

Beyond the enhanced platinum sensitivity observed in g*BRCA*-positive APC, therapeutic benefit in this subgroup has been further supported by the approval of poly(adenosine diphosphate–ribose) polymerase (PARP) inhibitors as maintenance therapy after platinum exposure.^12^ The evolving treatment paradigm—where platinum-based chemotherapy serves as an induction regimen preceding PARP inhibition—underscores the importance of understanding the kinetics and magnitude of antitumor activity associated with platinum-containing regimens. While several studies have reported superior radiologic responses among g*BRCA*-mutated patients treated with platinum-based chemotherapy,^6-8,14-18^ detailed insights into the depth and temporal dynamics of response during the treatment course remain limited.

This study aimed to elucidate the influence of g*BRCA* mutation status on the efficacy and safety of modified fluorouracil, leucovorin, irinotecan, and oxaliplatin (FOLFIRINOX; mFFX) in patients with APC. Furthermore, to explore the qualitative nature of treatment response, we performed an in-depth comparative analysis of radiologic and biological response kinetics among responders, thereby providing additional evidence for the mechanistic and therapeutic implications of g*BRCA* mutation status in the context of mFFX therapy.

## METHODS

### Patients and Genetic Testing for g*BRCA* Mutation

This multi-institutional, retrospective study included patients with APC treated at three tertiary referral centers. Medical records of patients with histologically confirmed, unresectable pancreatic cancer who received at least one cycle of mFFX between April 2018 and March 2023 were retrospectively reviewed. Screening for pathogenic g*BRCA1/2* variants was performed using either BRACAnalysis (Myriad Genetics Laboratories, Inc, Salt Lake, UT) or FoundationOne CDx (Foundation Medicine, Inc, Cambridge, MA). The determination of g*BRCA* mutation positivity was based exclusively on results obtained from BRACAnalysis. Patients who were not tested for g*BRCA* mutations during their therapeutic course were excluded from the study cohort.

### Treatment

The mFFX regimen comprised oxaliplatin 85 mg/m^2^, irinotecan 150 mg/m^2^, and l-leucovorin 200 mg/m^2^ administered intravenously, each once daily on day 1, followed by continuous intravenous infusion of 5-fluorouracil (5-FU) 2,400 mg/m^2^ once over 46 hours on day 1-3 every 2 weeks. Baseline computed tomography was performed before treatment initiation and repeated approximately every 2-3 months thereafter. Serum carbohydrate antigen 19-9 (CA19-9) concentrations were measured monthly throughout the therapeutic course. Treatment was continued until radiologically confirmed disease progression, development of intolerable adverse events (AEs), or patient withdrawal of consent, in accordance with RECIST version 1.1. Dose reductions or schedule delays were permitted at the discretion of the treating physician according to AE severity. The cumulative relative dose intensity (cRDI) was calculated as the average percentage ratio of the actual dose delivered to the planned cumulative doses of 5-FU, irinotecan, and oxaliplatin during the initial 3-month treatment period.

### Evaluation of Efficacy and Safety

The efficacy of mFFX was assessed by progression-free survival (PFS) and objective response rate (ORR). ORR was defined as the proportion of patients achieving either a complete (CR) or partial response (PR). Among patients demonstrating radiologic response, further analyses were conducted to compare the kinetics of tumor shrinkage and the normalization rate of serum CA19-9 within the first 6 months of treatment according to g*BRCA* mutation status. Safety was evaluated based on the incidence of AEs leading to dose reduction, treatment modification, or discontinuation.

### Ethical Standards

This study was conducted in accordance with the ethical principles outlined in the 1964 Declaration of Helsinki and its subsequent amendments. The study protocol was reviewed and approved by the institutional ethics committees of the participating centers: Kyushu Cancer Center (approval No.: 2023-06), Saiseikai Fukuoka General Hospital (approval No.: 2023-2), and Fukuoka Higashi Medical Center (approval No.: 2023-32). The requirement for informed consent was waived by the ethics committee because of the retrospective nature of the study, minimal risk to patients, and the use of deidentified data.

### Statistical Analysis

PFS and time to response were estimated using the Kaplan-Meier method, and group differences were evaluated using the log-rank test. The Cox proportional hazards model was used to calculate hazard ratios (HRs) and corresponding 95% CIs. Comparisons between two groups were performed using Student's *t* test for continuous variables and Fisher's exact test for categorical variables. For comparisons among three groups, one-way analysis of variance followed by Tukey's multiple comparison post hoc test was applied. Predictive factors for PFS and ORR were examined using multivariate Cox regression and logistic regression models, respectively. All reported *P* values were two-sided, with statistical significance defined as *P* < .05. All analyses were performed using Prism version 10.6 (GraphPad, San Diego, CA).

## RESULTS

### Patient Characteristics

A total of 178 patients received at least one cycle of mFFX, including 17 patients with g*BRCA* mutations and 161 without. Among the g*BRCA*-positive cohort, one patient harbored a g*BRCA1* mutation, 15 carried g*BRCA2* mutations, and one patient exhibited concurrent mutations in both g*BRCA1* and g*BRCA2*. Baseline characteristics are summarized in Table 1. The two groups demonstrated broadly comparable clinical and demographic features. Furthermore, the cRDI of 5-FU, irinotecan, and oxaliplatin during the first 3 months of treatment did not differ significantly between groups (Table 1).

**TABLE 1.** Patient Characteristics According to g*BRCA* Mutation Status

Characteristic	Total (N = 178)	g*BRCA*-Positive (n = 17)	g*BRCA*-Negative (n = 161)	*P*
Age, years, median (range)	63 (40-79)	59 (40-79)	63 (41-79)	.251
Sex, No. (%)				
Female	69 (39)	8 (47)	61 (38)	.602
Male	109 (61)	9 (53)	100 (62)	
Performance status, No. (%)				
0	70 (39)	8 (47)	62 (39)	.603
1-2	108 (61)	9 (53)	99 (61)	
Diabetes, No. (%)				
Yes	51 (29)	5 (29)	46 (29)	>.999
No	127 (71)	12 (71)	115 (71)	
Disease extent, No. (%)				
Locally advanced	27 (15)	2 (12)	25 (16)	>.999
Metastatic	151 (85)	15 (88)	136 (84)	
Recurrent, No. (%)				
Yes	23 (13)	1 (6)	22 (14)	.702
No	155 (87)	16 (94)	139 (86)	
Chemotherapy-naïve, No. (%)				
Yes	120 (67)	10 (59)	110 (68)	.426
No	58 (33)	7 (41)	51 (32)	
Time from diagnosis^a^ to mFFX, days, median (range)	27 (2-1,101)	36 (7-261)	26 (2-1,101)	.624
UGT1A1 poor metabolizer,^b^ No. (%)				
Yes	12 (7)	0 (0)	12 (7)	.609
No	166 (93)	17 (100)	149 (93)	
Location, No. (%)				
Head	93 (52)	12 (71)	81 (50)	.131
Body/tail	85 (48)	5 (29)	80 (50)	
Liver metastases, No. (%)				
Yes	94 (53)	9 (53)	85 (53)	>.999
No	84 (47)	8 (47)	76 (47)	
Bone metastases, No. (%)				
Yes	13 (7)	0 (0)	13 (8)	.617
No	165 (93)	17 (100)	148 (92)	
Ascites, No. (%)				
Yes	44 (25)	2 (12)	42 (26)	.248
No	134 (75)	15 (88)	119 (74)	
CA19-9 (U/mL), No. (%)				
Median (range)	633 (1-5,066,999)	2,408 (10-970,989)	624 (1-5,066,999)	.914
≤1,000	98 (55)	8 (47)	90 (56)	.610
≥1,001	80 (45)	9 (53)	71 (44)	
cRDI of 5-FU, %, median (range)	76 (33-102)	71 (41-101)	77 (33-102)	.462
cRDI of irinotecan, %, median (range)	74 (0-101)	71 (27-100)	74 (0-101)	.800
cRDI of oxaliplatin, %, median (range)	77 (29-102)	71 (47-100)	77 (29-102)	.547

Abbreviations: 5-FU, 5-fluorouracil; CA19-9, carbohydrate antigen 19-9; cRDI, cumulative relative dose intensity; FOLFIRINOX, fuluorouracil, folinic acid, irinotecan, and oxaliplatin; g*BRCA*, germline *BRCA*; mFFX, modified FOLFIRINOX.

^a^ Timing at diagnosis as unresectable or recurrent.

^b^ Includes homozygous UGT1A1*6 or UGT1A1*28 and heterozygous UGT1A1*6 and *28.

Patient disposition is detailed in Table 2. The median duration of mFFX therapy was significantly longer in the g*BRCA*-positive group than in the g*BRCA*-negative group (6.2 months [range, 2.2-22.5] *v* 3.8 months [range, 0.4-24.3]; *P* = .029). The most common reason for treatment discontinuation was disease progression, occurring in 41% and 75% of patients with and without g*BRCA* mutations, respectively. Discontinuation attributable to AEs (35% vs. 12%) and treatment switching (24% *v* 9%) was also more frequent among g*BRCA*-positive patients.

**TABLE 2.** Patient Disposition and Best Overall Response According to *gBRCA* Mutation Status

Factor	Total, No. (%)	g*BRCA*-Positive, No. (%)	g*BRCA*-Negative, No. (%)	Odds Ratio (95% CI)	*P*
Patient disposition					
Remaining on treatment	1 (2/178)	0 (0/17)	1 (2/161)	—	—
Discontinued treatment	99 (176/178)	100 (17/17)	99 (159/161)	—	—
Disease progression	72 (128/178)	41 (7/17)	75 (121/161)	—	—
Adverse events	15 (27/178)	35 (6/17)	13 (21/161)	—	—
Death	0 (0/178)	0 (0/17)	0 (0/161)	—	—
Withdrawal of consent	2 (3/178)	0 (0/17)	2 (3/161)	—	—
Switch treatment	10 (18/178)	24 (4/17)	9 (14/161)	—	—
Duration on mFFX, months, median (range)	4.4 (0.4-24.3)	6.2 (2.2-22.5)	3.8 (0.4-24.3)	—	.029
Best overall response					
Complete response	0 (0/178)	0 (0)	0 (0/161)	—	—
Partial response	38 (68/178)	82 (14/17)	34 (54/161)	—	—
Stable disease	16 (28/178)	6 (1/17)	17 (27/161)	—	—
Progressive disease	37 (66/178)	6 (1/17)	40 (65/161)	—	—
Not evaluated	9 (16/178)	6 (1/17)	9 (15/161)	—	—
Objective response rate	38 (68/178)	82 (14/17)	34 (54/161)	0.11 (0.03 to 0.39)	<.001
Normalization of CA19-9					
Baseline >370 U/mL	17 (18/106)	69 (9/13)	10 (9/93)	0.05 (0.01 to 0.18)	<.001
>1,000 U/mL	13 (10/80)	56 (5/9)	7 (5/71)	0.06 (0.01 to 0.32)	.001
>5,000 U/mL	8 (4/53)	50 (4/8)	0 (0/45)	0.00 (0.00 to 0.15)	<.001
>10,000 U/mL	7 (3/42)	43 (3/7)	0 (0/35)	0.00 (0.00 to 0.18)	.003

Abbreviations: CA19-9, carbohydrate antigen 19-9; FOLFIRINOX, fluorolracil, folinic acid, irinotecan, and oxaliplatin; g*BRCA*, germline *BRCA*; mFFX, modified FOLFIRINOX.

### Efficacy of mFFX: Factors Affecting PFS

**FIG A1.**
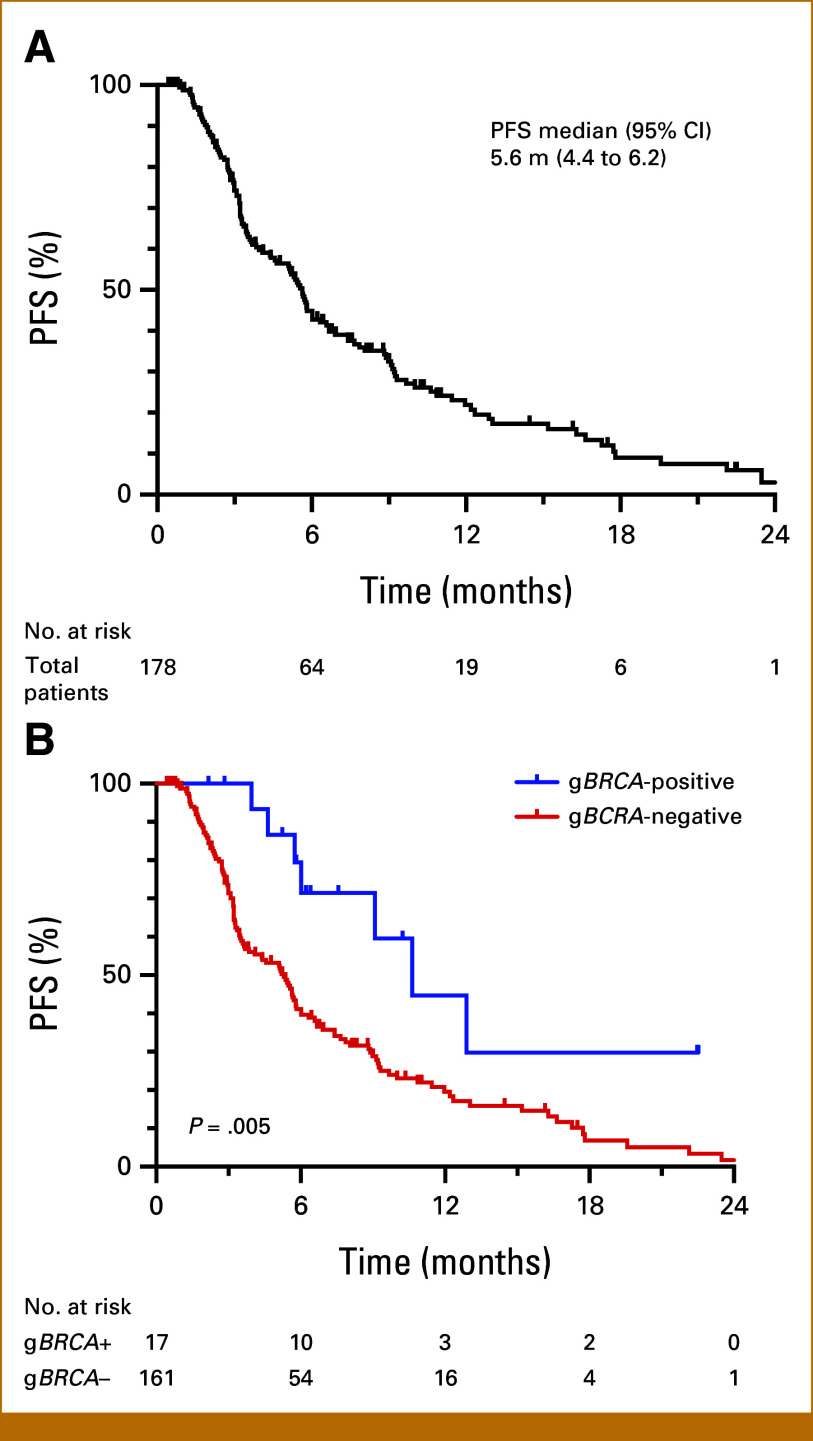
Kaplan-Meier curves for PFS. (A) PFS curve of all the enrolled patients. (B) PFS curves of all the enrolled patients according to g*BRCA* mutational status. g*BRCA*, germline *BRCA*; PFS, progression-free survival.

Across the entire cohort, the median PFS was 5.6 months (95% CI, 4.4 to 6.2; Appendix Fig A1A). Patients with g*BRCA* mutations exhibited a significantly prolonged PFS compared with those without mutations (10.6 months [95% CI, 6.0 to not available] *v* 5.3 months [95% CI, 3.6 to 5.8]; *P* = .005; Appendix Fig A1B).

Univariate analysis identified several clinical factors associated with longer PFS: Eastern Cooperative Oncology Group performance status (ECOG PS) 0 (7.9 *v* 4.6 months; *P* = .013), locally advanced disease (12.3 *v* 5.1 months; *P* < .001), and chemotherapy-naïve status (6.3 *v* 3.5 months; *P* = .003; Table 3). Conversely, shorter PFS was observed in patients with recurrent disease (3.0 *v* 5.8 months; *P* = .030), liver metastases (4.0 *v* 7.7 months; *P* = .012), and ascites (3.2 *v* 5.7 months; *P* = .033). In multivariate analysis, the absence of g*BRCA* mutation (HR, 3.65 [95% CI, 1.79 to 8.78]), metastatic disease (HR, 2.34 [95% CI, 1.15 to 5.27]), and previous chemotherapy (HR, 1.66 [95% CI, 1.09 to 2.50]) emerged as independent predictors of shorter PFS (Table 3).

**TABLE 3.** Univariate and Multivariate Analyses of PFS

Factor	No.	Univariate Analysis		Multivariate Analysis
		mPFS (95% CI)	*P*	HR (95% CI)
Age, years				
≤65	110	5.6 m (3.7 to 6.7)	.905	—
≥66	68	5.3 m (4.0 to 8.8)		—
Sex				
Female	69	7.4 m (4.6 to 9.4)	.528	—
Male	109	5.4 m (3.6 to 6.0)		—
Performance status				
0	70	7.9 m (5.7 to 10.0)	.013	Ref
1-2	108	4.6 m (3.3 to 5.6)		1.29 (0.88 to 1.91)
Diabetes				
Yes	51	3.7 m (3.2 to 6.7)	.663	—
No	127	5.7 m (5.1 to 7.4)		—
Disease extent				
Locally advanced	27	12.3 m (9.7 to NA)	<.001	Ref
Metastatic	151	5.1 m (3.5 to 5.8)		2.34 (1.15 to 5.27)
Recurrent				
Yes	23	3.0 m (2.5 to 3.7)	.030	1.42 (0.79 to 2.46)
No	155	5.8 m (5.2 to 7.4)		Ref
Chemotherapy-naïve				
Yes	120	6.3 m (5.5 to 8.8)	.003	Ref
No	58	3.5 m (2.8 to 5.3)		1.66 (1.09 to 2.50)
UGT1A1 poor metabolizer^a^				
Yes	12	9.0 m (3.5 to 9.3)	.928	—
No	166	5.5 m (4.4 to 6.0)		—
g*BRCA* mutation				
Yes	17	10.6 m (6.0 to NA)	.005	Ref
No	161	5.3 m (3.6 to 5.8)		3.65 (1.79 to 8.78)
Location				
Head	93	5.6 m (3.9 to 6.7)	.875	—
Body/tail	85	5.7 m (3.6 to 7.4)		—
Liver metastases				
Yes	94	4.0 m (3.2 to 5.6)	.012	1.40 (0.93 to 2.13)
No	84	7.7 m (5.6 to 10.6)		Ref
Bone metastases				
Yes	13	3.5 m (2.2 to 7.7)	.420	—
No	165	5.7 m (4.6 to 6.7)		—
Ascites				
Yes	44	3.2 m (3.0 to 6.3)	.033	1.11 (0.72 to 1.67)
No	134	5.7 m (5.1 to 7.7)		Ref
CA19-9 (U/mL)				
≤1,000	98	5.8 m (4.6 to 7.9)	.648	—
≥1,001	80	5.1 m (3.5 to 6.0)		—

Abbreviations: CA19-9, carbohydrate antigen 19-9; g*BRCA*, germline *BRCA*; HR, hazard ratio; mPFS, median progression-free survival; NA, not available; Ref, reference.

^a^ Includes homozygous UGT1A1*6 or UGT1A1*28 and heterozygous UGT1A1*6 and *28.

### Efficacy of mFFX: Factors Affecting ORR

No patients achieved CR; however, PR was observed in 14 g*BRCA*-positive patients (82%) and 54 g*BRCA*-negative patients (34%), as shown in Table 2. The ORR was significantly higher in g*BRCA*-positive patients than in g*BRCA*-negative patients (82% *v* 34%; odds ratio [OR], 0.11 [95% CI, 0.03 to 0.39]; *P* < .001).

Univariate analysis demonstrated superior ORR among patients with ECOG PS 0 (57% *v* 32%; *P* = .002) and locally advanced disease (83% *v* 35%; *P* < .001) and those who were chemotherapy-naïve (51% *v* 22%; *P* < .001; Table 4). Conversely, patients with recurrent tumors showed markedly reduced ORR (14% *v* 46%; *P* = .004). Multivariate analysis identified the absence of g*BRCA* mutation (OR, 0.05 [95% CI, 0.01 to 0.22]), metastatic disease (OR, 0.16 [95% CI, 0.04 to 0.50]), tumor recurrence (OR, 0.27 [95% CI, 0.06 to 0.95]), previous chemotherapy (OR, 0.29 [95% CI, 0.10 to 0.71]), and poorer PS (OR, 0.44 [95% CI, 0.29 to 0.96]) as independent predictors of reduced ORR (Table 4).

**TABLE 4.** Univariate and Multivariate Analyses of Objective Response Rate

Factors	No.	Univariate Analysis		Multivariate Analysis
		ORR, %	*P*	HR (95% CI)
Age, years				
≤65	100	46	.195	—
≥66	62	35		—
Sex				
Female	62	44	.870	—
Male	100	41		—
Performance status				
0	65	57	.002	Ref
1-2	97	32		0.44 (0.29 to 0.96)
Diabetes				
Yes	46	30	.078	—
No	116	47		—
Disease extent				
Locally advanced	23	83	<.001	Ref
Metastatic	139	35		0.16 (0.04 to 0.50)
Recurrent				
Yes	22	14	.004	0.27 (0.06 to 0.95)
No	140	46		Ref
Chemotherapy-naïve				
Yes	111	51	<.001	Ref
No	51	22		0.29 (0.10 to 0.71)
UGT1A1 poor metabolizer^a^				
Yes	10	40	>.999	—
No	152	42		—
g*BRCA* mutation				
Yes	16	88	<.001	Ref
No	146	37		0.05 (0.01 to 0.22)
Location				
Head	82	43	.875	—
Body/tail	80	41		—
Liver metastases				
Yes	86	38	.342	—
No	76	46		—
Bone metastases				
Yes	13	31	.560	—
No	149	43		—
Ascites				
Yes	38	29	.090	—
No	124	46		—
CA19-9 (U/mL)				
≤1,000	93	41	.750	—
≥1,001	69	43		—

Abbreviations: CA19-9, carbohydrate 19-9; g*BRCA*, germline *BRCA*; HR, hazard ratio; ORR, objective response rate; Ref, reference.

^a^ Includes homozygous UGT1A1*6 or UGT1A1*28 and heterozygous UGT1A1*6 and *28.

### Efficacy of mFFX: Changes in Serum CA19-9 Levels

The median time to normalization of serum CA19-9 was 4.4 months (range, 2.1-10.8) in the g*BRCA*-positive group and 4.2 months (range, 2.1-6.9) in the g*BRCA*-negative group. Among the 106 patients whose baseline CA19-9 levels exceeded 370 U/mL (10 times the upper limit of normal [ULN]), the normalization rate was significantly higher in g*BRCA*-positive patients than in those without g*BRCA* mutations (69% *v* 10%; OR, 0.05 [95% CI, 0.01 to 0.18; Table 2). Comparable trends were observed when higher baseline CA19-9 thresholds were applied (Table 2).

### Kinetics of Radiologic and Biological Responses According to g*BRCA* Mutation Status

**FIG 1.**
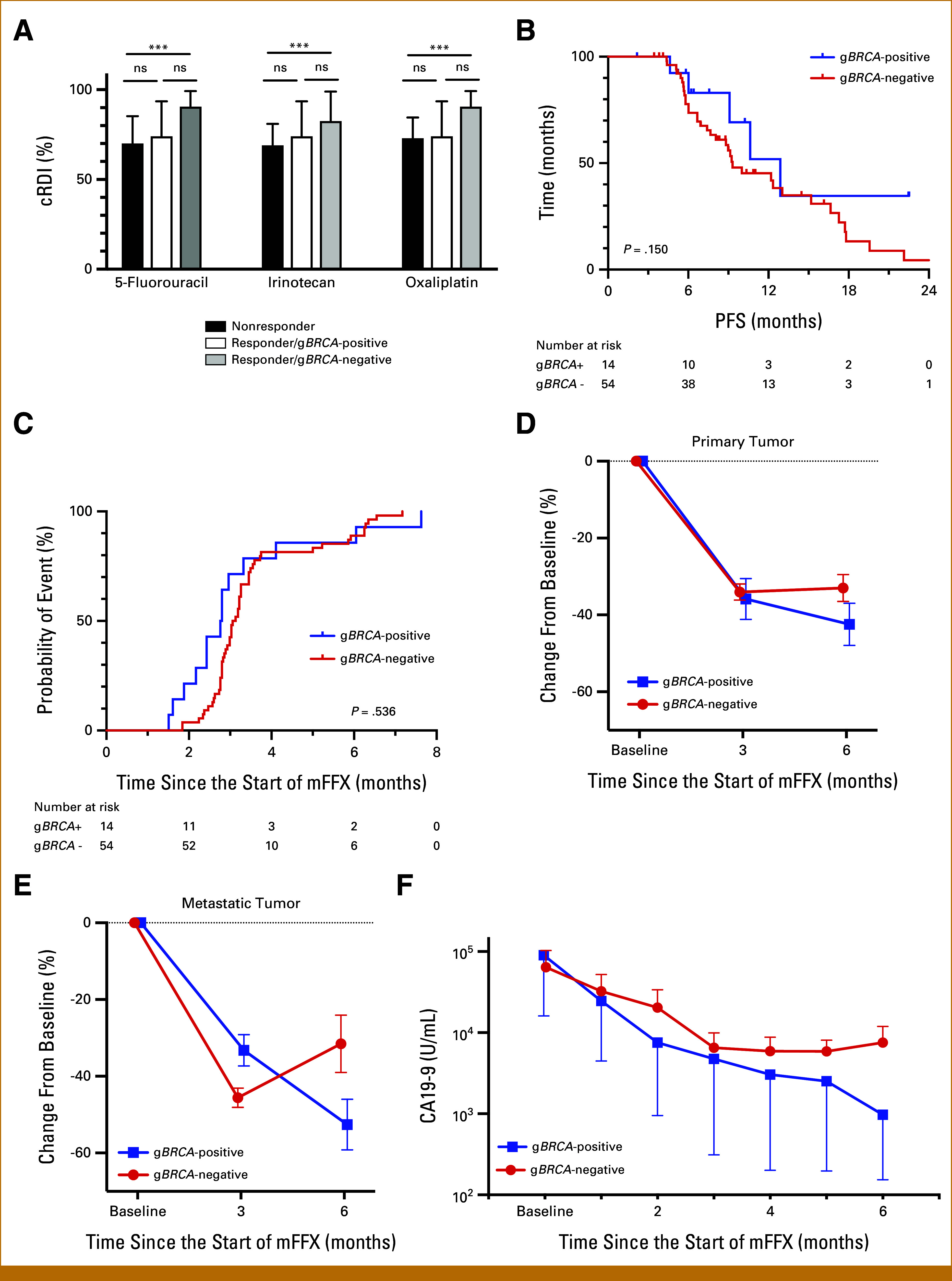
Effect of g*BRCA* mutation status on mFFX efficacy among responders. (A) Comparison of cRDI for each agent between nonresponders and responders with or without g*BRCA* mutation (median with interquartile range). (B) PFS curves of responders according to g*BRCA* mutation status. (C) Kaplan-Meier analysis of time to response by g*BRCA* mutation status. Changes in the size of (D) primary tumors, (E) metastatic tumors, and (F) serum CA19-9 levels within the first 6 months of treatment, according to g*BRCA* mutation status (mean ± SEM). CA19-9, carbohydrate antigen 19-9; cRDI, cumulative relative dose intensity; FOLFIRINOX, fluorouracil, folinic acid, irinotecan, and oxaliplatin; g*BRCA*, germline *BRCA*; mFFX, modified FOLFIRINOX; PFS, progression-free survival; SEM, standard error of the mean. ***P < .001.

Patients who achieved PR were classified as responders and included in a detailed exploratory analysis. Of the 68 responders, 14 patients were g*BRCA*-positive and 54 were g*BRCA*-negative. Baseline characteristics were comparable between the two groups, except for a significantly higher proportion of patients with previous chemotherapy exposure in the g*BRCA*-positive group (43% *v* 9%; *P* = .007; Appendix Table A1). No significant differences were observed in the cRDI of 5-FU, irinotecan, or oxaliplatin between g*BRCA*-positive and g*BRCA*-negative responders, whereas cRDI was significantly lower in nonresponders compared with that in g*BRCA*-negative responders (Appendix Table A1, Fig 1A), possibly reflecting the significantly shorter duration of exposure to mFFX in nonresponders than in g*BRCA*-negative responders (2.9 months [range, 0.4-23.5] *v* 8.1 months [range, 3.5-24.3]; *P* < .001).

Among responders, median PFS did not differ significantly between g*BRCA*-positive and g*BRCA*-negative groups (12.9 months [95% CI, 6.02 to not available] *v* 9.3 months [95% CI, 7.7 to 13.3]; *P* = .150; Fig 1B). Similarly, the median time to response was comparable between groups (2.8 months [95% CI, 2.2 to 3.0] *v* 3.1 months [95% CI, 2.9 to 3.3]; *P* = .536; Fig 1C).

Radiologic response dynamics are illustrated in Figures 1D and 1E. At 3 months, the degree of tumor shrinkage in both primary and metastatic lesions was similar across the two groups. By 6 months, however, continuous tumor regression was observed in 82% (9 of 11) of primary tumors and 100% (10 of 10) of metastatic lesions among g*BRCA*-positive patients. By contrast, tumor regrowth was evident at 6 months in 40% (19 of 48) of primary tumors and 38% (12 of 32) of metastatic lesions in g*BRCA*-negative patients (Figs 1D and 1E).

Longitudinal assessment of biological response revealed sustained reductions in median CA19-9 levels throughout the initial 6 months of treatment in g*BRCA*-positive patients (Fig 1F). In g*BRCA*-negative patients, CA19-9 levels declined during the first 3 months but subsequently plateaued (Fig 1F). Among 38 g*BRCA*-negative patients with baseline CA19-9 levels above the ULN, re-elevation of CA19-9 was observed in 17 cases (45%) during the first 6 months.

### Subgroup Analysis

**FIG 2.**
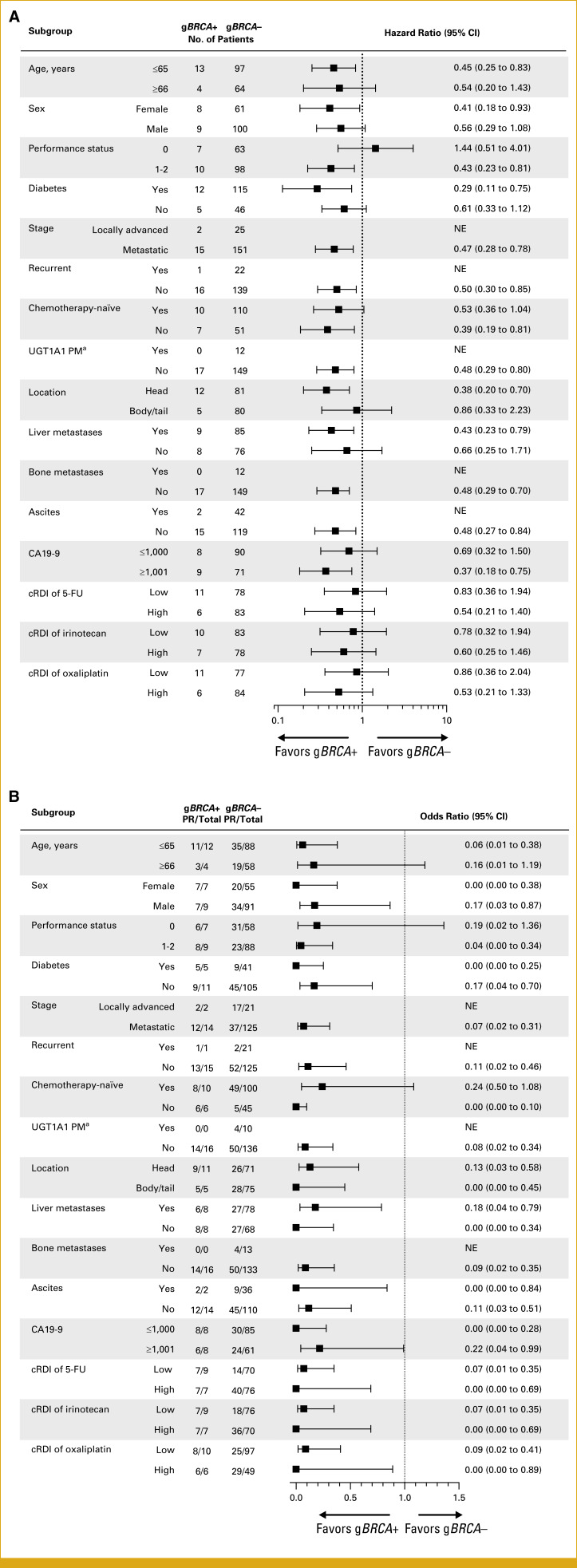
Forest plots of PFS and ORR by subgroup. Forest plots showing subgroup analyses by baseline characteristics for (A) PFS in all patients and (B) ORR in those who underwent at least one radiologic assessment. Dots represent (A) hazard ratios or (B) odds ratios; whiskers indicate 95% CI. ^a^Includes homozygous UGT1A1*6 or UGT1A1*28 and heterozygous UGT1A1*6 and *28. 5-FU, 5-fluorouracil; CA19-9, carbohydrate antigen 19-9; cRDI, cumulative relative dose intensity; g*BRCA*, germline *BRCA*; NE, not evaluated; ORR, objective response rate; PFS, progression-free survival; PM, poor metabolizer; PR, partial response.

Subgroup analyses revealed that most patient subgroups demonstrated superior PFS in the g*BRCA*-positive cohort compared with that in the g*BRCA*-negative cohort (Fig 2A). An exception was observed among patients with PS 0, who exhibited slightly longer PFS in the g*BRCA*-negative group; this finding is likely attributable to the limited sample size within this subgroup. The benefit of g*BRCA* mutation status on ORR was generally consistent across all examined subgroups (Fig 2B). Although several subgroups did not reach statistical significance, this was considered to be at least partially as a result of the relatively small number of patients within these categories.

### Safety

More than 80% of patients required at least one dose modification as a result of AEs (Appendix Table A2). The overall safety profile was comparable between the g*BRCA*-positive and g*BRCA*-negative groups. However, discontinuation of mFFX attributable to AEs occurred in 35% of g*BRCA*-positive patients, which was significantly higher than that in the g*BRCA*-negative group (13%; *P* = .026). Peripheral neuropathy represented the most frequent AE leading to treatment discontinuation among g*BRCA*-positive patients and occurred at a markedly higher rate than in g*BRCA*-negative patients (29% *v* 1%; *P* < .001) Other AE-related discontinuations were infrequent and did not differ significantly between groups (Appendix Table A2).

## DISCUSSION

The present study demonstrated that patients with APC harboring g*BRCA* mutations exhibited markedly superior responses to mFFX, including prolonged PFS and higher ORR and CA19-9 normalization rate compared with g*BRCA*-negative patients. In addition, our current study showed, to our knowledge, for the first time that increased platinum sensitivity with g*BRCA* mutation in APC was characterized by sustained radiologic and biological response within the first 6 months, which was not observed in g*BRCA*-negative responders. Among all analyzed variables, g*BRCA* mutation status emerged as the strongest independent predictor of both PFS and ORR. Furthermore, subgroup analyses confirmed that the clinical advantage associated with g*BRCA* positivity was consistently observed across most baseline characteristics.

These findings align closely with recent Japanese studies evaluating the efficacy of platinum-containing chemotherapy in *BRCA*-mutated pancreatic cancer. Sudo et al^6^ reported data from the GOZILA study, a nationwide ctDNA sequencing analysis, demonstrating significantly longer PFS after platinum-based therapy in g*BRCA*-positive patients than in g*BRCA*-negative patients (9.3 *v* 4.3 months; HR, 0.55, *P* = .027). Similarly, an analysis of a national genomic profiling database including Japanese patients revealed a significantly longer time to treatment failure among those with *BRCA/PALB2* mutations compared with patients harboring other HRD-related mutations or no HRD alterations (9.2 *v* 6.3 *v* 4.7 months; *P* < .01).^7^ Multiple additional reports have confirmed that platinum-containing regimens yield prolonged PFS in HRD-positive patients regardless of the disease stage^8,9^ and that ORRs of 56%-89% in this subgroup significantly exceed those observed in HRD-negative patients (14%-32%).^6-8,14-18^ Collectively, these results strongly corroborate our findings, reinforcing the substantial therapeutic advantage conferred by g*BRCA* mutations in patients treated with mFFX.

Notably, in our cohort, the PFS advantage associated with gBRCA mutations diminished when analyses were restricted to responders within the g*BRCA-*negative group. This suggests that enhanced platinum sensitivity in HRD-mutated tumors is characterized not only by a longer treatment duration but also by a greater depth or degree of tumor response. This interpretation is supported by the numerically higher rate of biological response observed in the g*BRCA*-positive cohort. Previous studies have highlighted the clinical value of CA19-9 kinetics as a surrogate marker of treatment response during FFX therapy^19,20^ and as a predictor of surgical outcomes.^21,22^ For example, Kim et al^19^ reported that among 242 patients (188 with locally advanced or metastatic disease) treated with first-line FFX, 24% achieved CA19-9 normalization at 8 weeks, which correlated significantly with both PFS and OS. In the neoadjuvant setting, approximately 30% of patients achieved CA19-9 normalization after 4 months of preoperative FFX,^20^ and such normalization has consistently been associated with improved surgical outcomes.^21,22^ Furthermore, a subset of HRD-positive patients has been reported to achieve either radiologic or pathologic CR,^14,23^ an outcome rarely observed among platinum-treated HRD-negative tumors.^3,24^ Taken together, these findings indicate that the superior platinum sensitivity conferred by g*BRCA* mutations is closely linked to a deeper and more sustained treatment response, both radiologically and biologically. Our results therefore underscore the critical importance of routine g*BRCA* screening in patients with APC to guide optimal therapeutic strategies, particularly for those eligible for platinum-based chemotherapy.

To date, no previous studies have specifically examined differences in the safety profile of mFFX according to g*BRCA* mutation status and findings regarding chemotherapy toxicity have been inconsistent. *BRCA1* mutations have been associated with an increased risk of febrile neutropenia in breast cancer,^25^ whereas no significant effect on hematologic toxicity has been reported in ovarian cancer.^26^ The spectrum and frequency of AEs observed in the present study were comparable with those reported in previous studies of mFFX in unselected populations.^3,24^ The significantly higher rate of AE-related treatment discontinuation among g*BRCA*-positive patients observed in our cohort may be explained by the availability of subsequent treatment with PARP inhibitor maintenance therapy. Indeed, four of five g*BRCA*-positive patients who discontinued mFFX because of peripheral neuropathy subsequently switched to PARP inhibitor maintenance therapy. The expectation of transition to maintenance therapy might have led both physicians and patients to discontinue mFFX earlier, potentially overestimating the severity of self-reported AEs, particularly peripheral neuropathy. Moreover, longer duration of treatment exposure observed in g*BRCA*-positive patients may exacerbate the severity of several cumulative toxicities, represented by peripheral neuropathy, which can also result in significantly higher rate of AE-induced treatment failure in g*BRCA*-positive patients than in the g*BRCA*-negative group. Given that the phase III POLO trial demonstrated greater survival benefit from olaparib maintenance among patients who received more than 6 months of first-line platinum-based chemotherapy,^27^ optimal management of AEs is essential to prevent premature discontinuation of mFFX and thereby sustain the survival advantage conferred by both mFFX and PARP inhibitor therapy in this population.

This study has several limitations inherent to its retrospective design, including potential selection and information biases. The relatively small sample size, particularly the limited number of g*BRCA*-positive patients, might have led to an overestimation of the efficacy and safety outcomes associated with mFFX, and thus, the results should be considered exploratory. In addition, the lack of zygosity calling precluded confirmation of true biallelic *BRCA* loss, which–although more common in g*BRCA*-mutant tumors–is particularly difficult to assess in pancreatic cancer because of the low tumor cellularity and purity, potentially introducing biological and technical heterogeneity that might have attenuated the prolongation of PFS beyond the effect of limited sample size.^28^ Furthermore, as the primary aim of this study was to investigate differences in treatment response, overall survival was not evaluated. Although a number of previous studies have consistently demonstrated a significant survival benefit associated with g*BRCA* mutations in APC treated with platinum-based chemotherapy,^7-9,16,29,30^ further large-scale prospective studies are warranted to validate and expand upon these findings.

In conclusion, to our knowledge, this is the first study to evaluate the effect of g*BRCA* mutation status on both radiologic and biological responses to mFFX in APC, alongside an assessment of treatment safety. The superiority associated with g*BRCA* mutations was characterized by a more profound and sustained therapeutic response during the first 6 months of treatment, as evidenced by continuous tumor shrinkage and marked declines in serum CA19-9 levels. Our findings underscore the clinical importance of universal g*BRCA* screening in patients with APC. Determining g*BRCA* mutation status can provide critical information to guide therapeutic decision making, optimize treatment sequencing, and ultimately improve long-term outcomes in this highly lethal malignancy.

## APPENDIX

**TABLE A1.** Characteristics of Responders

Characteristic	g*BRCA*-Positive, No. (%)	g*BRCA*-Negative, No. (%)	*P*
Age, years, median (range)	60 (40-79)	62 (41-79)	.585
Sex, No. (%)			
Female	7 (50)	20 (37)	.541
Male	7 (50)	34 (63)	
Performance status, No. (%)			
0	6 (43)	31 (57)	.378
1-2	8 (57)	23 (43)	
Diabetes, No. (%)			
Yes	5 (36)	9 (17)	.144
No	9 (64)	45 (83)	
Disease extent, No. (%)			
Locally advanced	2 (14)	17 (31)	.319
Metastatic	12 (86)	37 (69)	
Recurrent, No. (%)			
Yes	1 (7)	2 (4)	.505
No	13 (93)	52 (96)	
Chemotherapy-naïve			
Yes	8(57)	49(91)	.007
No	6(43)	5(9)	
Time from diagnosis^a^ to mFFX, days, median (range)	34 (7-261)	21 (2-741)	.295
UGT1A1 poor metabolizer,^b^ No. (%)			
Yes	0 (0)	4 (7)	.574
No	14 (100)	50 (93)	
Location, No. (%)			
Head	9 (64)	26 (48)	.372
Body/tail	5(36)	28 (52)	
Liver metastases, No. (%)			
Yes	6 (43)	27 (50)	.767
No	8 (57)	27 (50)	
Bone metastases, No. (%)			
Yes	0 (0)	4 (7)	.574
No	14 (100)	50 (93)	
Ascites, No. (%)			
Yes	2 (14)	9 (17)	>.999
No	12 (86)	45 (83)	
CA19-9 (U/mL), median (range)	732 (10-970,989)	626 (2-1,487,000)	.570
≤1,000	6 (43)	24 (44)	>.999
≥1,001	8 (57)	30 (56)	
cRDI of 5-FU, %, median (range)	72 (45-96)	81 (50-101)	.370
cRDI of irinotecan, %, median (range)	72 (15-96)	78 (30-100)	.433
cRDI of oxaliplatin, %, median (range)	72 (53-96)	81 (42-101)	.584

Abbreviations: 5-FU, 5-fluorouracil; CA19-9, carbohydrate antigen 19-9; cRDI, cumulative relative dose intensity; FOLFIRINOX, fluorouracil, folinic acid, irinotecan, and oxaliplatin; g*BRCA*, germline *BRCA*; mFFX, modified FOLFIRINOX.

^a^ Timing at diagnosis as unresectable or recurrent.

^b^ Includes homozygous UGT1A1*6 or UGT1A1*28 and heterozygous UGT1A1*6 and *28.

**TABLE A2.** Comparison of Safety Profiles According to g*BRCA* Mutation Status

Factor	Total (N = 178), %	*gBRCA*-Positive (n = 17), %	*gBRCA*-Negative (n = 161), %	*P*
AEs related to dose modification	84	88	84	>.999
Fatigue	47	59	45	.317
Neutropenia	39	41	39	>.999
Anorexia/nausea	24	35	22	.239
Physician decision	16	12	16	>.999
Diarrhea	15	6	16	.474
Peripheral neuropathy	13	29	12	.069
Infection	11	0	12	.224
UGT1A1 poor metaboliser	6	0	6	.601
Febrile neutropenia	5	0	6	>.999
Thrombocytopenia	3	0	4	>.999
AEs related to treatment discontinuation	15	35	13	.026
Fatigue	7	6	7	>.999
Peripheral neuropathy	3	29	1	<.001
Anorexia/nausea	3	6	2	.398
Neutropenia	2	0	2	>.999
Febrile neutropenia	1	0	1	>.999
Diarrhea	1	0	1	>.999
Infection	1	0	1	>.999
Physician decision	1	0	1	>.999
Use of prophylactic G-CSF	8	12	8	.640
Time from the start of mFFX, months, median (range)	1.5 (0.7-5.6)	1.9 (0.9-2.9)	1.5 (0.7-5.6)	.675

Abbreviations: AEs, adverse events; FOLFIRINOX, fluorouracil, folinic acid, irinotecan, and oxaliplatin; g*BRCA*, germline *BRCA*; G-CSF, granulocyte colony-stimulating factor; mFFX, modified FOLFIRINOX.

## References

[1] Rahib L, Smith BD, Aizenberg R, et al: Projecting cancer incidence and deaths to 2030: The unexpected burden of thyroid, liver, and pancreas cancers in the United States. Cancer Res 74:2913-2921, 2014 24840647 10.1158/0008-5472.CAN-14-0155

[2] Von Hoff DD, Ervin T, Arena FP, et al: Increased survival in pancreatic cancer with nab-paclitaxel plus gemcitabine. N Engl J Med 369:1691-1703, 2013 24131140 10.1056/NEJMoa1304369 PMC4631139

[3] Conroy T, Desseigne F, Ychou M, et al: FOLFIRINOX versus gemcitabine for metastatic pancreatic cancer. N Engl J Med 364:1817-1825, 2011 21561347 10.1056/NEJMoa1011923

[4] Turner N, Tutt A, Ashworth A: Hallmarks of 'BRCAness' in sporadic cancers. Nat Rev Cancer 4:814-819, 2004 15510162 10.1038/nrc1457

[5] Narod SA, Foulkes WD: BRCA1 and BRCA2: 1994 and beyond. Nat Rev Cancer 4:665-676, 2004 15343273 10.1038/nrc1431

[6] Sudo K, Nakamura Y, Ueno M, et al: Clinical utility of BRCA and ATM mutation status in circulating tumour DNA for treatment selection in advanced pancreatic cancer. Br J Cancer 131:1237-1245, 2024 39198618 10.1038/s41416-024-02834-0 PMC11443054

[7] Ishigaki K, Tokito Y, Takahara N, et al: Association between homologous recombination deficiency and time to treatment failure to platinum-based chemotherapy for pancreatic cancer by using the C-CAT database. J Gastroenterol 60:247-256, 2025 39570378 10.1007/s00535-024-02173-0 PMC11794350

[8] Wattenberg MM, Asch D, Yu S, et al: Platinum response characteristics of patients with pancreatic ductal adenocarcinoma and a germline BRCA1, BRCA2 or PALB2 mutation. Br J Cancer 122:333-339, 2020 31787751 10.1038/s41416-019-0582-7 PMC7000723

[9] Park W, Chen J, Chou JF, et al: Genomic methods identify homologous recombination deficiency in pancreas adenocarcinoma and optimize treatment selection. Clin Cancer Res 26:3239-3247, 2020 32444418 10.1158/1078-0432.CCR-20-0418 PMC7380542

[10] Golan T, Kindler HL, Park JO, et al: Geographic and ethnic heterogeneity of germline BRCA1 or BRCA2 mutation prevalence among patients with metastatic pancreatic cancer screened for entry into the POLO trial. J Clin Oncol 38:1442-1454, 2020 32073954 10.1200/JCO.19.01890

[11] Goldstein JB, Zhao L, Wang X, et al: Germline DNA sequencing reveals novel mutations predictive of overall survival in a cohort of patients with pancreatic cancer. Clin Cancer Res 26:1385-1394, 2020 31871297 10.1158/1078-0432.CCR-19-0224

[12] Golan T, Hammel P, Reni M, et al: Maintenance olaparib for germline BRCA-mutated metastatic pancreatic cancer. N Engl J Med 381:317-327, 2019 31157963 10.1056/NEJMoa1903387 PMC6810605

[13] Tempero MA: NCCN guidelines updates: Pancreatic cancer. J Natl Compr Canc Netw 17:603-605, 2019 31117041 10.6004/jnccn.2019.5007

[14] Orsi G, Di Marco M, Cavaliere A, et al: Chemotherapy toxicity and activity in patients with pancreatic ductal adenocarcinoma and germline BRCA1-2 pathogenic variants (gBRCA1-2pv): A multicenter survey. ESMO Open 6:100238, 2021 34392104 10.1016/j.esmoop.2021.100238 PMC8371213

[15] Park JH, Jo JH, Jang SI, et al: BRCA 1/2 germline mutation predicts the treatment response of FOLFIRINOX with pancreatic ductal adenocarcinoma in Korean patients. Cancers (Basel) 14:236, 2022 35008403 10.3390/cancers14010236 PMC8750183

[16] Golan T, O'Kane GM, Denroche RE, et al: Genomic features and classification of homologous recombination deficient pancreatic ductal adenocarcinoma. Gastroenterology 160:2119-2132 e9, 2021 33524400 10.1053/j.gastro.2021.01.220

[17] Kubo T, Muramatsu J, Arihara Y, et al: Clinical characterization of patients with gBRCA1/2 mutation-positive unresectable pancreatic cancer: A multicenter prospective study. Jpn J Clin Oncol 54:47-53, 2024 37791389 10.1093/jjco/hyad131 PMC10773213

[18] Reiss KA, Yu S, Judy R, et al: Retrospective survival analysis of patients with advanced pancreatic ductal adenocarcinoma and germline BRCA or PALB2 mutations. JCO Precis Oncol 10.1200/PO.17.00152 10.1200/PO.17.00152 35135099

[19] Kim SS, Kim S, Jo JH, et al: Early response evaluation using CT and CA 19-9 in patients with pancreatic cancer of all stages undergoing first-line FOLFIRINOX treatment. Pancreatology 25:377-384, 2025 40050184 10.1016/j.pan.2025.02.007

[20] Thalji SZ, Kamgar M, George B, et al: CA19-9 response to first-line neoadjuvant FOLFIRINOX and second-line Gemcitabine/nab-paclitaxel for patients with operable pancreatic cancer. Ann Surg Oncol 30:3013-3021, 2023 36788189 10.1245/s10434-022-13055-1

[21] Yun WG, Han Y, Cho YJ, et al: In neoadjuvant FOLFIRINOX chemotherapy for pancreatic ductal adenocarcinoma, which response is the more reliable indicator for prognosis, radiologic or biochemical? Ann Surg Oncol 31:1336-1346, 2024 37991581 10.1245/s10434-023-14532-x

[22] Servin-Rojas M, Fong ZV, Fernandez-Del Castillo C, et al: Tumor size reduction and serum carbohydrate antigen 19-9 kinetics after neoadjuvant FOLFIRINOX in patients with pancreatic ductal adenocarcinoma. Surgery 175:471-476, 2024 37949693 10.1016/j.surg.2023.09.041

[23] Golan T, Barenboim A, Lahat G, et al: Increased rate of complete pathologic response after neoadjuvant FOLFIRINOX for BRCA mutation carriers with borderline resectable pancreatic cancer. Ann Surg Oncol 27:3963-3970, 2020 32314163 10.1245/s10434-020-08469-8

[24] Kobayashi N, Omae K, Horita Y, et al: FOLFIRINOX as second-line chemotherapy for advanced pancreatic cancer: A subset analysis of data from a nationwide multicenter observational study in Japan. Pancreatology 20:1519-1525, 2020 32972834 10.1016/j.pan.2020.07.006

[25] Friedlaender A, Vuilleumier A, Viassolo V, et al: BRCA1/BRCA2 germline mutations and chemotherapy-related hematological toxicity in breast cancer patients. Breast Cancer Res Treat 174:775-783, 2019 30635808 10.1007/s10549-018-05127-2

[26] Weitzner O, Yagur Y, Kadan Y, et al: Chemotherapy toxicity in BRCA mutation carriers undergoing first-line platinum-based chemotherapy. Oncologist 24:e1471-e1475, 2019 31346131 10.1634/theoncologist.2019-0272 PMC6975939

[27] Kindler HL, Hammel P, Reni M, et al: Overall survival results from the POLO trial: A phase III study of active maintenance olaparib versus placebo for germline BRCA-mutated metastatic pancreatic cancer. J Clin Oncol 40:3929-3939, 2022 35834777 10.1200/JCO.21.01604 PMC10476841

[28] Jonsson P, Bandlamudi C, Cheng ML, et al: Tumour lineage shapes BRCA-mediated phenotypes. Nature 571:576-579, 2019 31292550 10.1038/s41586-019-1382-1 PMC7048239

[29] Golan T, Kanji ZS, Epelbaum R, et al: Overall survival and clinical characteristics of pancreatic cancer in BRCA mutation carriers. Br J Cancer 111:1132-1138, 2014 25072261 10.1038/bjc.2014.418 PMC4453851

[30] Smith AL, Wong C, Cuggia A, et al: Reflex testing for germline BRCA1, BRCA2, PALB2, and ATM mutations in pancreatic cancer: Mutation prevalence and clinical outcomes from two Canadian research registries. JCO Precis Oncol 10.1200/PO.17.00098 10.1200/PO.17.00098 35135108

